# X‐ray CT metal artifact reduction using neural attenuation field prior

**DOI:** 10.1002/mp.17859

**Published:** 2025-04-30

**Authors:** Jooho Lee, Seongjun Kim, Junhyun Ahn, Adam S. Wang, Jongduk Baek

**Affiliations:** ^1^ Department of Artificial Intelligence Yonsei University Seoul Republic of Korea; ^2^ School of Integrated Technology Yonsei University Seoul Republic of Korea; ^3^ Department of Radiology Stanford University California USA

**Keywords:** 3D deep learning, metal artifact reduction, neural field, sinogram inpainting, x‐ray CT

## Abstract

**Background:**

The presence of metal objects in computed tomography (CT) imaging introduces severe artifacts that degrade image quality and hinder accurate diagnosis. While several deep learning‐based metal artifact reduction (MAR) methods have been proposed, they often exhibit poor performance on unseen data and require large datasets to train neural networks.

**Purpose:**

In this work, we propose a sinogram inpainting method for metal artifact reduction that leverages a neural attenuation field (NAF) as a prior. This new method, dubbed NAFMAR, operates in a self‐supervised manner by optimizing a model‐based neural field, thus eliminating the need for large training datasets.

**Methods:**

NAF is optimized to generate prior images, which are then used to inpaint metal traces in the original sinogram. To address the corruption of x‐ray projections caused by metal objects, a 3D forward projection of the original corrupted image is performed to identify metal traces. Consequently, NAF is optimized using a metal trace‐masked ray sampling strategy that selectively utilizes uncorrupted rays to supervise the network. Moreover, a metal‐aware loss function is proposed to prioritize metal‐associated regions during optimization, thereby enhancing the network to learn more informed representations of anatomical features. After optimization, the NAF images are rendered to generate NAF prior images, which serve as priors to correct original projections through interpolation. Experiments are conducted to compare NAFMAR with other prior‐based inpainting MAR methods.

**Results:**

The proposed method provides an accurate prior without requiring extensive datasets. Images corrected using NAFMAR showed sharp features and preserved anatomical structures. Our comprehensive evaluation, involving simulated dental CT and clinical pelvic CT images, demonstrated the effectiveness of NAF prior compared to other prior information, including the linear interpolation and data‐driven convolutional neural networks (CNNs). NAFMAR outperformed all compared baselines in terms of structural similarity index measure (SSIM) values, and its peak signal‐to‐noise ratio (PSNR) value was comparable to that of the dual‐domain CNN method.

**Conclusions:**

NAFMAR presents an effective, high‐fidelity solution for metal artifact reduction in 3D tomographic imaging without the need for large datasets.

## 1 INTRODUCTION

Metal artifact reduction (MAR) is one of the most challenging and long‐standing problems in computed tomography (CT).^1^ The high attenuation of metallic objects often leads to strong attenuation or even complete absorption of x‐rays passing through them, resulting in corrupted projection data. In addition, factors such as noise, beam hardening, scatter, and nonlinear partial volume effects further complicate this problem.^2^ Consequently, the reconstructed CT images using this incomplete projection data exhibit severe streaks, commonly known as metal artifacts. Metal artifacts are especially troublesome in orthopedics and dentistry, where metal implants obstruct the visualization of neighboring anatomical structures, thereby hindering accurate diagnosis and treatment planning.^3^

To reduce metal artifacts and to improve CT image quality, several MAR methods have been proposed.^4^, ^5^, ^6^ Notably, sinogram inpainting methods have been widely employed.^7^ The underlying idea of these methods is to consider any projection data affected by metals (the so‐called metal trace) as missing data. Then, an interpolation^8^ or incorporation of prior knowledge^9^, ^10^ is utilized to correct the original sinogram by inpainting surrogate data into the metal trace. Recently, driven by the remarkable advancements in artificial intelligence (AI),^11^ various AI‐based MAR methods have been introduced by leveraging supervised learning frameworks.^12^, ^13^ For example, convolutional neural networks (CNNs) have demonstrated promising performance in reducing severe artifacts.^14^, ^15^ Researchers have extended these image processing techniques across various domains, including the sinogram domain,^16^, ^17^ the image domain,^18^ and dual‐domain approaches^19^, ^20^ to improve MAR performance. However, despite their promising results, the practical application of these supervised learning‐based methods is constrained by their heavy reliance on large paired datasets, which are often infeasible to acquire in clinical practice. Unsupervised methods, on the other hand, have been proposed to address the issue of limited paired training data.^21^, ^22^ Nonetheless, many of these methods still depend on large, clean datasets to learn the prior distribution. Additionally, the generalizability of these data‐driven methods across diverse imaging protocols remains a significant concern in medical imaging.^23^

To overcome these challenges, self‐supervised learning has emerged as a promising alternative.^24^, ^25^ In particular, implicit neural representation (INR) offers a novel model‐driven paradigm for solving inverse problems^26^ such as tomographic imaging.^27^, ^28^ One notable example is neural attenuation field (NAF)^29^ that optimizes a multi‐layer perceptron (MLP) to represent attenuation coefficient values of CT‐scanned objects. This is achieved by minimizing the error between the network's synthesized and measured projections, thereby maximizing multi‐view data consistency. While recent work has investigated neural fields for limited‐data CT reconstruction,^30^ the problem of metal artifacts remains largely unexplored. Furthermore, the majority of current MAR methods are designed to handle 2D imaging scenarios such as parallel‐beam or fan‐beam CT. Therefore, an effective 3D MAR solution for cone‐beam CT (CBCT) has not yet been fully explored.

In this work, we propose a novel MAR method for CBCT imaging that leverages an NAF prior to inpaint original projections. We show that NAF offers a more accurate and feasible prior compared to CNN, even without requiring large datasets for training. To achieve this, we first formulate tomographic reconstruction as a neural field. Then, to optimize the neural field, we propose a metal trace‐masked ray sampling strategy to carefully select uncorrupted x‐rays, thus optimizing the network without being affected by corrupted rays. In addition, we design a metal‐aware loss to enhance the network's ability to learn accurate representations near metal implants. This loss function serves as an attention to induce strong inductive bias and improves the quality of missing data completions. Lastly, we leverage the output of the neural field as a prior to inpaint surrogate data into the metal trace, which effectively addresses the spectral bias^31^ of neural fields and provides high‐fidelity reconstructions. Combined, we call our method NAFMAR, which operates in a self‐supervised manner, requiring only the measurements as input. Experimental results on simulated and clinical datasets demonstrate NAFMAR's superiority over compared baselines in reducing metal artifacts while preserving anatomical structure. In summary, our main contributions are:

1. We propose a sinogram inpainting method for CT metal artifact reduction that leverages a neural attenuation field prior, thus eliminating the need for large training data.
2. We introduce a metal trace‐masked ray sampling and metal‐aware loss to optimize the network by selectively utilizing reliable information from metal‐corrupted projections.
3. We demonstrate the effectiveness of our model‐driven neural attenuation field prior in comparison to other priors, such as data‐driven convolutional neural networks.

## 2 RELATED WORK

### 2.1 Metal artifact reduction

Traditional methods for MAR can be broadly categorized into sinogram inpainting, iterative, and physics‐based methods. One of the earliest sinogram inpainting methods employed linear interpolation (LI)^7^ to correct metal‐trace regions, which was further enhanced by normalized MAR (NMAR),^8^ where a prior image was incorporated to improve interpolation accuracy. Additionally, an image‐based frequency splitting approach was proposed in frequency split MAR (FSMAR)^10^ to better preserve sharp features. On the other hand, iterative methods^4^ have been explored by considering image regularizers like total variation^32^ to refine CT image quality. Researchers have also developed a polychromatic data acquisition model to more accurately reflect the underlying physics of imaging, which has proven effective in reducing beam hardening artifacts by optimizing the likelihood of measurements.^33^

Recently, deep learning‐based MAR methods have demonstrated superior performance compared to traditional methods.^34^ For example, CNNs were widely applied to reduce metal artifacts,^18^ and this was later extended to other network architectures such as Vision Transformer (ViT).^35^ Current deep learning methods can be grouped into sinogram domain, image domain, and dual domain approaches. Sinogram domain approaches use neural networks to restore metal‐corrupted sinogram data, followed by filtered‐back projection (FBP) to produce reconstructions.^17^ Image domain approaches employ networks to directly learn the mapping function from metal‐corrupted images to clean images.^36^ Here, residual learning and adversarial training have been explored to leverage rich features.^37^ Lastly, dual domain networks utilize networks in both domains with differentiable FBP to improve MAR quality.^19^, ^38^

Despite their promise, these methods often face challenges in their robustness, as deep networks are known to be vulnerable to small perturbations.^39^ Moreover, the lack of paired data (i.e., metal‐corrupted and clean images) poses significant difficulties in practice. In this work, we show that current top‐performing methods perform poorly on unseen datasets, producing unwanted artifacts and exhibiting unpredictable behavior. We also demonstrate that a self‐supervised strategy of using neural field priors can achieve results that are superior or at least comparable to those of supervised learning‐based methods.

### 2.2 Neural fields

Neural field techniques have gained increasing popularity in the field of 3D vision.^40^ In particular, neural radiance fields (NeRF)^26^ have emerged as a powerful method of representing 3D scenes.^41^ Inspired by NeRF, researchers have proposed neural rendering approaches for various tasks such as shape or surface reconstruction.^42^, ^43^ In the context of medical imaging, several works adapted a differentiable projector to reconstruct CT images as a continuous representation.^25^, ^27^, ^28^ More recently, these works have been extended to 3D reconstructions by employing hierarchical representations^30^ and neural attenuation fields.^29^, ^44^ However, most of these methods are tailored for sparse‐view or limited‐angle CT, where the projection data is incomplete compared to full‐view scanning.

When metal objects are present in the field of measurement, x‐ray projection itself becomes corrupted, making these naive approaches unreliable. While the recently proposed Polyner optimizes a neural field with a polychromatic forward model to alleviate nonlinear metal artifacts, it fails to address photon starvation, where photons can be completely attenuated.^45^ In such cases, its performance drops significantly due to missing projection data. Moreover, its assumption on the prior knowledge of the energy spectrum of incident x‐ray beam may not always hold in clinical practice.^46^ Finally, considering that most current MAR methods^45^, ^47^ are designed for 2D imaging scenarios, further advancement in 3D neural fields is needed.

Meanwhile, the spectral bias^31^ of neural fields for 3D tomographic imaging has also not been thoroughly investigated in previous studies.^29^, ^44^ In this work, we show that the neural field struggles to represent high‐frequency variations when reconstructing a 3D tomographic volume. Specifically, we demonstrate that while neural fields can accurately capture structural information, their noise texture tends to be relatively blurred. To address this problem within the MAR framework, we leverage the approach of using neural field renderings to obtain prior images. As a result, the proposed method establishes a new, effective 3D MAR baseline that reconstructs both structural and fine details with high fidelity.

## 3 METHODS

**FIGURE 1:**
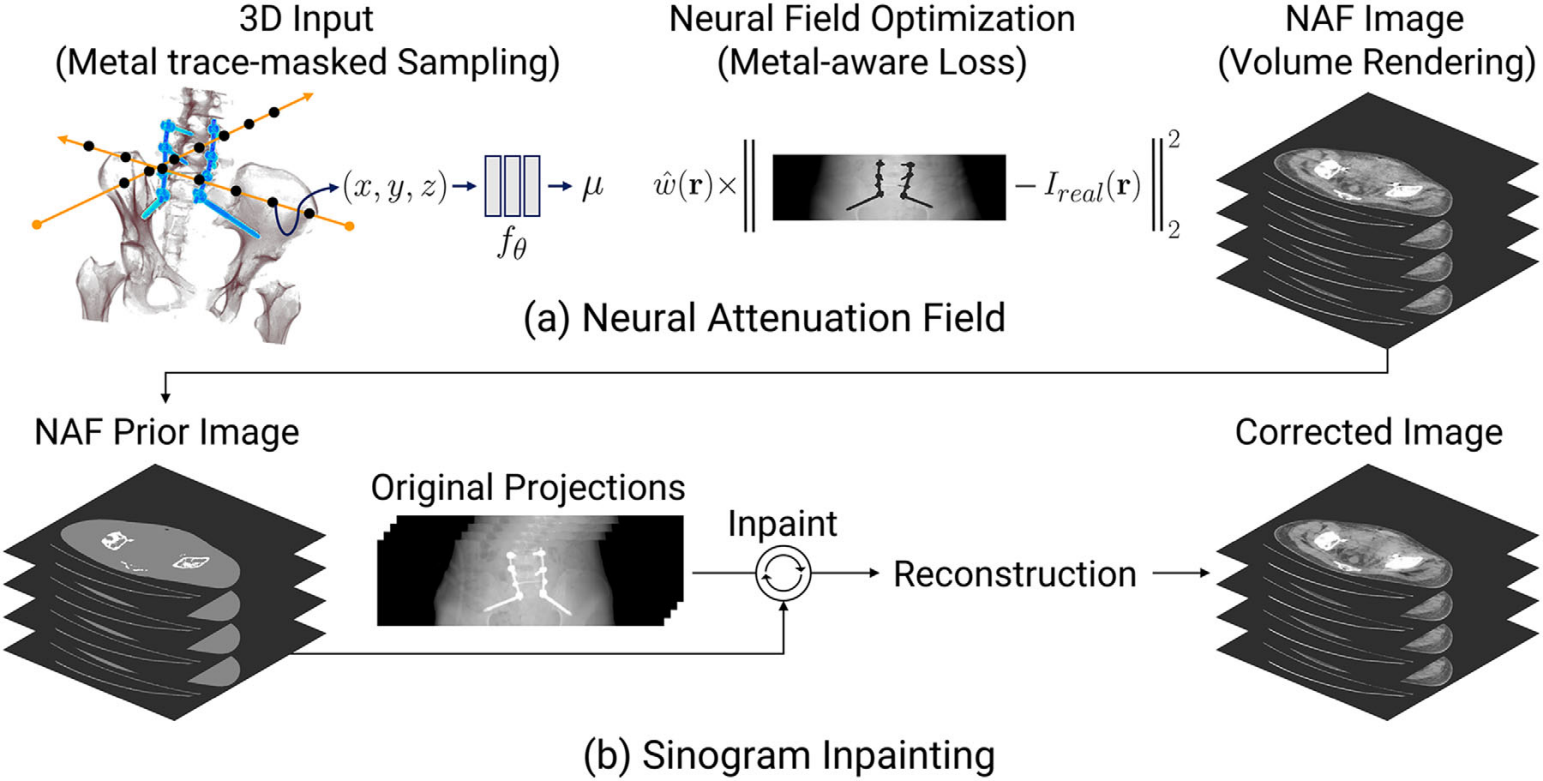
Overview of NAFMAR, comprising (a) NAF optimization and (b) sinogram inpainting using NAF prior. MAR, metal artifact reduction; NAF, neural attenuation field.

In this work, we present NAFMAR, a neural field prior‐based sinogram inpainting method for CT metal artifact reduction. To obtain prior information, our method formulates 3D tomographic reconstruction as an optimization problem that involves a neural attenuation field. More specifically, we optimize a deep fully‐connected neural network to represent the attenuation coefficient distribution of the CT scanned object. Since x‐ray projections are corrupted within the presence of metal objects, we propose a metal trace‐masked ray sampling strategy coupled with the metal‐aware loss to enable accurate reconstruction of 3D neural renderings. Finally, by utilizing these renderings, we generate prior images to correct the original metal‐corrupted projections and achieve high‐fidelity metal artifact reduction. Figure 1 visualizes this overall pipeline.

### 3.1 Neural attenuation field

Implicit neural representation for CT imaging is referred to as NAF,^29^ as it represents the attenuation coefficient distribution of the CT‐scanned object. Mathematically, NAF can be expressed as a 3D vector‐valued function:

(1) $$f_{\theta }:\;\left(x,y,z\right)\to \mu$$

where $f_{\theta }$ denotes the neural network with weights θ that maps 3D input coordinates to their corresponding attenuation coefficients μ∈R. According to Beer–Lambert's law, the intensity of ray penetrating through an object is reduced by the exponential integration of the attenuation coefficients along its path. Hence, we can synthesize the predicted projection of ray $r\left(t\right)=o+td\in R^{3}$ as:

(2) $$I_{pred}\left(r\right)=I_{0}\cdot \text{exp}\left(-\int_{t_{n}}^{t_{f}}\mu \left(r\left(t\right)\right)dt\right)$$

where $I_{0}$ is the initial ray intensity, and $t_{n}$ and $t_{f}$ represent the given near and far bounds of the ray, respectively. In practice, we approximate this integral in discrete form:

(3) $$I=I_{0}\cdot \exp\left(-\sum_{i=1}^{N}\mu_{i}\delta_{i}\right)$$

where N denotes the number of discretized samples and $\delta_{i}=∥t_{i+1}-t_{i}∥$ is the distance between adjacent points.

In addition, as this process is differentiable, we can use gradient descent to optimize the network $f_{\theta }$ by minimizing the discrepancy (i.e., squared error) between the predicted and measured projections:

(4) $$L_{\text{RECON}}\left(\theta ,R\right)=\sum_{r\in R}\left|\right|I_{pred}\left(r\right)-I_{real}\left(r\right){\left|\right|}_{2}^{2}$$

where R indicates a set of input rays and $I_{real}$ are measurements (i.e., x‐ray projections). Once the network is fully optimized, we can render the tomographic volume to obtain reconstructions.

### 3.2 Metal trace‐masked ray sampling

We aim to optimize the neural attenuation field from metal‐corrupted measurements. However, unlike previous studies^25^, ^29^ that utilize all rays within the measurements, the presence of metal objects complicates our problem. This is because the x‐ray projection itself becomes corrupted when the corresponding ray intersects with metallic objects.^8^ These unreliable values, commonly termed the metal trace, are the main contributors to the metal artifacts in the reconstructed CT images. Therefore, these values should be excluded during our ray sampling process to ensure the fidelity of reconstruction.

To this end, we propose a metal trace‐masked ray sampling procedure that allows us to effectively supervise the network using only the reliable (i.e., uncorrupted) x‐ray information. To do so, we first segment the metal‐only images from the original FBP images by using a thresholding method.^14^ We then forward‐project these segmented metal‐only images to identify the metal‐corrupted regions (i.e., metal trace). Here, the projections with the value greater than zero are regarded as in the metal trace $T_{metal}$. Leveraging this information, we define our reliable set of rays $R_{r}$ as:

(5) $$R_{r}=\left\{r|r\in R\;\text{and}\;r\notin T_{metal}\right\}\;$$

Consequently, as this refined set of rays $R_{r}$ excludes metal‐corrupted projections, we can now optimize a high‐fidelity neural attenuation field using the loss function $L_{\text{RECON}}\left(\theta ,R_{r}\right)$, which enforces data consistency:

(6) $$L_{\text{RECON}}\left(\theta ,R_{r}\right)=\sum_{r\in R_{r}}\left|\right|I_{pred}\left(r\right)-I_{real}\left(r\right){\left|\right|}_{2}^{2}$$

In this study, we adopt the network $f_{\theta }$ consisting of 4 fully‐connected layers with ReLU activations and a width of 64 channels per layer. Additionally, to enhance the high‐frequency representation capacity of the MLP network, the input 3D coordinates are first fed into a multi‐resolution hash encoding^48^ before being processed by fully‐connected layers.

### 3.3 Metal‐aware loss

We have described the core components for optimizing a neural attenuation field for metal artifact reduction. However, we observed that these components are not sufficient to obtain high‐quality 3D reconstructions (see Section 5.3 for more discussion). We believe this is because the network $f_{\theta }$ lacks supervision for metal‐trace samples, resulting in ill‐posed scenarios. Therefore, we introduce several improvements to enhance the quality of reconstruction.

First, we design a metal‐aware loss based on our observations that the network is prone to generating suboptimal renderings, particularly in regions near metals. Recent studies have shown that modulating ray contributions during training can enhance neural renderings.^49^, ^50^ Building upon these findings, we incorporate metal awareness into our NAF framework. The following steps are taken to achieve this. We first calculate the Euclidean distance for each ray r from the sampled point (i.e., where an x‐ray is detected in detector space) to the nearest metal object region, defined as:

(7) $$d\left(r\right)=\min_{j}\left|\right|c_{r}-c_{{\text{metal}}_{j}}{\left|\right|}_{2}\;.$$

Here, $c_{r}$ and $c_{{\text{metal}}_{j}}$ are the 2D detector coordinates associated with each sampled ray r and the j‐th metal trace sample, respectively. Then, the set of metal‐aware rays, denoted as $R_{m}$ is defined based on these distances:

(8) $$R_{m}=\left\{r|r\in R_{r}\;\text{and}\;d\left(r\right)<d_{\text{th}}\right\}$$

where $d_{\text{th}}$ determines the boundary of metal‐aware region. Consequently, for the rays within this metal‐aware region, we assign weights that are inversely proportional to their distances from metal objects:

(9) w(r)=exp(−λ·d(r))

where λ denotes the decay factor. These weights act as attention, guiding the network to focus on metal‐associated regions by assigning higher weights to the ray samples near metal objects. Finally, by incorporating these weights, we define the metal‐aware loss as:

(10) $$L_{\text{MA}}\left(\theta ,R_{m}\right)=\sum_{r\in R_{m}}\hat{w}\left(r\right)\cdot \left|\right|I_{pred}\left(r\right)-I_{real}\left(r\right){\left|\right|}_{2}^{2}\;$$

Note that the normalization step of $\hat{w}\left(r\right)=w\left(r\right)/\max_{r^{\prime }}w\left(r^{\prime }\right)$ is applied to maintain a consistent scale across the weights. In our experiments, we set $d_{\text{th}}=20$ and λ=0.01.

### 3.4 Total loss

In summary, the total loss we optimize in each iteration is:

(11) $$L=L_{\text{RECON}}\left(\theta ,R_{r}\right)+\lambda_{M}L_{\text{MA}}\left(\theta ,R_{m}\right)\;$$

where we empirically set $\lambda_{M}=1$ and randomly sample ray directions for $R_{r}$ and $R_{m}$ within the 3D CT geometry.

**FIGURE 2:**
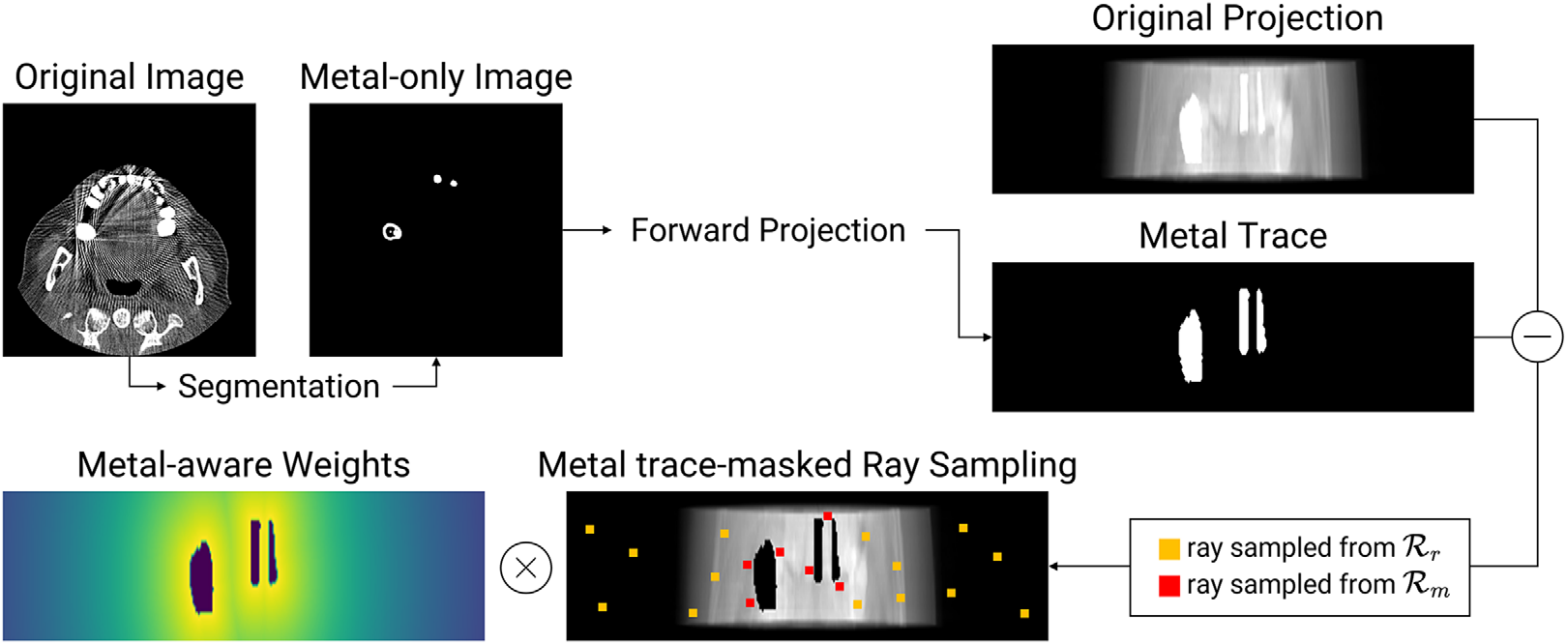
Illustration of the ray sampling process and metal‐aware loss. The symbol (−) denotes the exclusion of metal trace values from the original projection during ray sampling. The symbol (×) represents pixel‐wise multiplication used to calculate the metal‐aware loss.

Furthermore, we implement an area‐based sampling strategy to balance the number of rays sampled from $R_{r}$ and $R_{m}$ within each batch. Given a batch size, the number of ray samples for each set is determined by the relative sizes of their sampling areas in the detector space. For instance, if the entire detector space is four times larger than the metal‐aware region, four times more rays are sampled from $R_{r}$ than from $R_{m}$ to ensure a balanced distribution. Moreover, to prevent redundant ray sampling, rays sampled from $R_{m}$ are excluded when sampling from $R_{r}$. Figure 2 illustrates this sampling procedure along with the weighting scheme for metal‐aware loss.

### 3.5 Sinogram inpainting

While the network effectively reconstructs artifact‐reduced CT images by leveraging the proposed ray sampling strategy and loss function, residual artifacts may persist. Furthermore, despite the use of positional encoding,^48^ we found that the network struggles to accurately represent high‐frequency variations such as texture, mainly due to the spectral bias of deep neural networks.^31^ This is consistent with recent works on NeRF,^51^ which shows that NeRF inherently suffers from blurring artifacts in high resolution rendering. We hypothesize that this concern is more pronounced in tomographic imaging due to its extensive 3D sampling space encompassing nearly all points, including those internal to the object within the scan field of view. See Section 6 for more discussion.

In the context of CT metal artifact reduction, prior‐based sinogram inpainting (completion) method often offers a promising solution. This involves replacing the metal‐corrupted segments in the original sinogram with prior information. For example, previous studies considered using adaptive filtering^5^ or CNN^14^ to obtain such prior. Building upon these findings, we leverage sinogram inpainting strategy to address the imperfection of neural attenuation fields and to improve the quality of final reconstruction.

**FIGURE 3:**
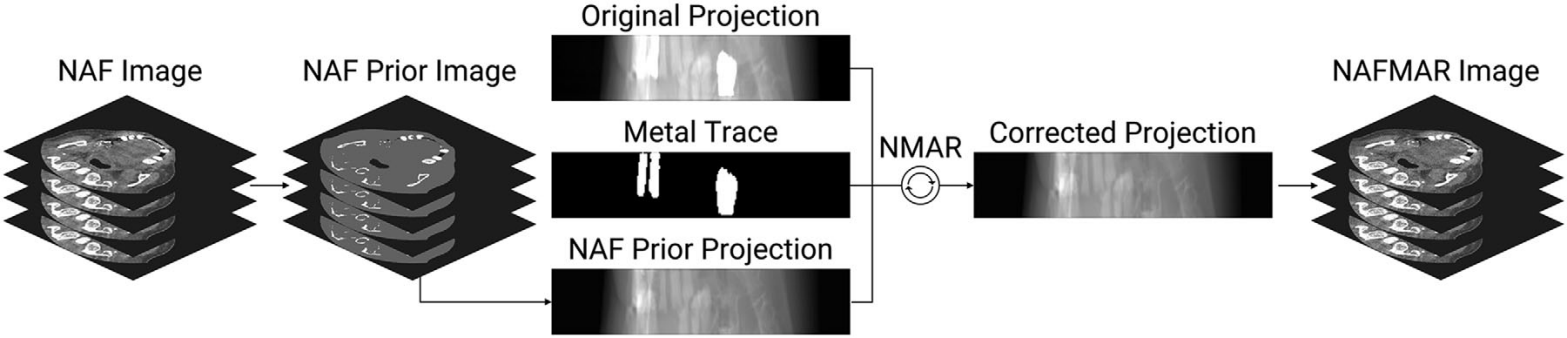
Illustration of the sinogram inpainting process.

To do so, the following steps are taken: First, we obtain the artifact‐reduced volumetric image by querying corresponding 3D input coordinates to the optimized network. We refer to this image as the NAF image. Next, we generate a prior image from the NAF image using the *k*‐means clustering‐based segmentation.^14^ We now refer to this prior image as the NAF prior image for the following. In this work, we employ a 3‐class clustering, with each class generally representing bone, soft tissue, and air. Consistent with Meyer et al.,^8^ the segmented air and soft tissue are then set to ‐1000 and 0 Hounsfield unit (HU), respectively, while pixels representing bone are retained at their original values. Once the NAF prior image is generated, we forward‐project this image to obtain NAF prior projections. The original corrupted projections are then normalized by dividing them pixel‐wise by the NAF prior projections. Here, a small positive value ($10^{-7}$) was used as a threshold to avoid division by zero. Next, interpolation is applied along the metal trace using the normalized projections. For simplicity, we used linear interpolation per detector row, though more advanced methods, such as bicubic interpolation, can be employed considering both row and column information. Finally, the corrected projections are obtained by denormalizing the interpolated projections. At last, the FBP reconstruction^52^ of these corrected projections yields our final result. In the corrected image, we reinsert the original metal values to visualize the implants based on the segmentation performed earlier. We illustrate this sinogram inpainting process in Figure 3.

## 4 EXPERIMENTS

### 4.1 Simulated dataset

**FIGURE 4:**
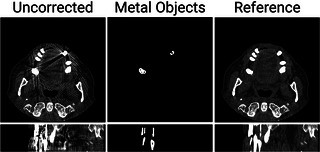
Example axial (top) and sagittal (bottom) slices from the simulated dataset.

We used dental CT images from the cancer imaging archive (TCIA)^53^ and manually inserted metal implants to generate our simulated CT dataset. Similar to previous studies,^20^, ^54^ we followed the metal artifact simulation method described in Zhang et al.^14^ To this end, we first segmented the metal‐inserted images into soft tissue, bone, and metal components. We then simulated polychromatic x‐ray projections from each of these segmented components, assuming a 120 kVp tube voltage. Specifically, we simulated 12 energy levels of x‐ray source, ranging from 10 to 120 keV, with a step size of 10 keV. The number of incident photons was set to $10^{6}$ to generate Poisson noise. Water beam hardening correction^55^ was applied to all generated sinograms and the corresponding images were reconstructed using 3D filtered back projection (FDK) with a standard reconstruction kernel. The metal implants were considered as gold, and x‐ray attenuation was simulated using data from the National Institute of Standards and Technology (NIST).^56^ We also varied the number (from 2 to 5), shape, and size of metal inserts to consider diverse clinical imaging scenarios. For CBCT geometry, the source‐to‐iso‐center and source‐to‐detector distances were set to 560 and 1000 mm, respectively. The 2D detector consisted of 512 columns and 64 rows, with each pixel measuring 1.286 mm. The reconstructed CBCT images had a matrix size of 512 × 512 × 100 voxels, with individual voxels sized at 0.4 mm × 0.4 mm × 0.4 mm. In Figure 4, we show sample images from our simulated CT dataset.

### 4.2 Clinical dataset

We further qualitatively compared the MAR performance on a clinical dataset. Specifically, we used CT images with dimensions of 512×512×187 voxels from the CLINIC‐metal dataset^57^ to assess clinical feasibility. Similar to Yu et al.,^54^ the clinical metal implants were segmented using a threshold of 2000 HU. However, one can also adapt an AI segmentation model^58^ to further improve segmentation accuracy.

### 4.3 Baselines

We compared the proposed method with five established sinogram inpainting‐based MAR approaches: LI,^7^ NMAR,^8^ FSNMAR,^10^ CNNMAR,^14^ and DSCMAR.^54^ It should be noted that CNNMAR and DSCMAR utilize data‐driven priors from paired data supervision, contrasting with our self‐supervised approach that operates solely on measurement data. Nevertheless, we included these methods as supervised learning counterparts using image domain and dual domains, respectively. For CNNMAR and DSCMAR training, we used 1551 pairs of metal‐free and metal‐corrupted CT images from the simulated dataset. Specifically, 2D slices containing metal artifacts were selected from the simulated CBCT data of six patients, excluding artifact‐free slices. We then evaluated the performance on both simulated and clinical datasets. Additionally, we compared prior images generated by NMAR, CNNMAR, and our method to evaluate the effectiveness of our model‐driven prior against data‐driven priors. DSCMAR was excluded from this comparison as it does not explicitly generate a prior image.

### 4.4 Metrics

For the simulated dataset, we use peak signal‐to‐noise ratio (PSNR) and structural similarity index measure (SSIM)^59^ to quantitatively evaluate MAR performance. For the clinical dataset, only visual assessments are provided due to the absence of ground truth. Given the reconstructed image x and the reference image y, PSNR is defined by:

(12) $$\text{PSNR}\left(x,y\right)=10\cdot \log_{10}\left(\frac{{\text{MAX}}_{y}^{2}}{\text{MSE}(x,y)}\right)$$

where MSE(x,y) is the mean squared error between x and y, and ${\mathrm{MAX}}_{y}$ denotes the maximum pixel value of y.

On the other hand, as a perceptual image quality metric, SSIM is defined as:

(13) $$\text{SSIM}\left(x,y\right)=\frac{\left(2\mu_{x}\mu_{y}+C_{1}\right)\left(2\sigma_{xy}+C_{2}\right)}{\left(\mu_{x}^{2}+\mu_{y}^{2}+C_{1}\right)\left(\sigma_{x}^{2}+\sigma_{y}^{2}+C_{2}\right)}$$

where $\mu_{x}$ and $\mu_{y}$ denote the mean values of images x and y, respectively; $\sigma_{x}$ and $\sigma_{y}$ represent the standard deviations of x and y; and $\sigma_{xy}$ is the covariance between x and y. The positive constants $C_{1}$ and $C_{2}$ are used to avoid a null denominator. For PSNR and SSIM calculations, metal implants were masked to ensure a fair assessment.

### 4.5 Implementation details

We build our framework based on the NAF codebase.^29^ In our experiments, we use a batch size of 256 rays, with each ray sampled at 576 coordinates. For hash encoding,^48^ we set the hash table size to $2^{16}$ and the number of feature dimensions to 4, while maintaining the original setup from ref. [48] for other hyperparameters. For further details about hash encoding, we refer readers to Müller et al.^48^ We use the Adam optimizer^60^ with an initial learning rate of $1\times 10^{-3}$, which decays by a factor of 0.5 every 50 epochs. The network consists of 4.5 million trainable parameters, which correspond to approximately 17 MB of memory. During training, GPU usage was approximately 4 GB, though this may vary depending on batch size. All models are trained for 150 epochs (77K steps) on a single NVIDIA RTX 3090 GPU, with a total training time of approximately 15 min. Prior image generation and 3D forward projection take 0.6 s, followed by sinogram inpainting (3.1 s), and FBP reconstruction (3.5 s), resulting in a total testing runtime of 7.2 s. We used TIGRE^61^ to perform 3D forward projection and back projection on Intel i5 CPU.

## 5 RESULTS

**FIGURE 5:**
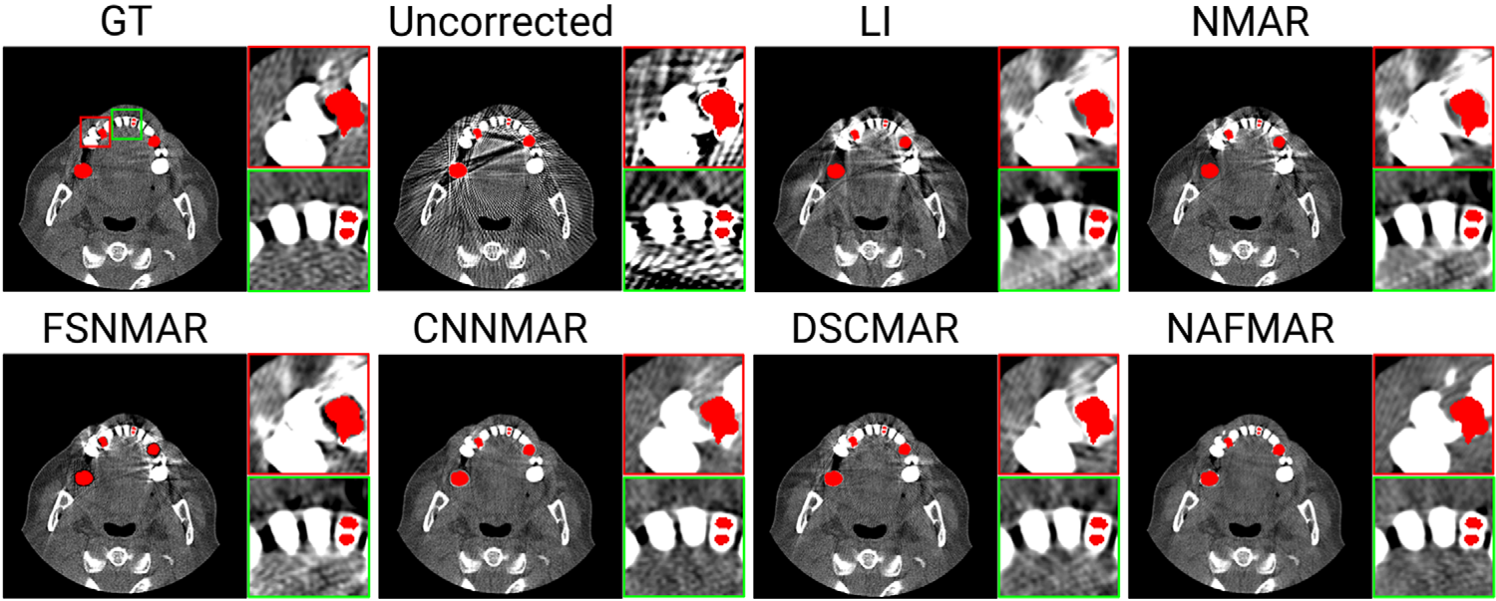
Qualitative comparisons on the simulated dataset with five metal implants. The display window is [−300, 700] Hounsfield unit, and the metal objects are colored red.

**FIGURE 6:**
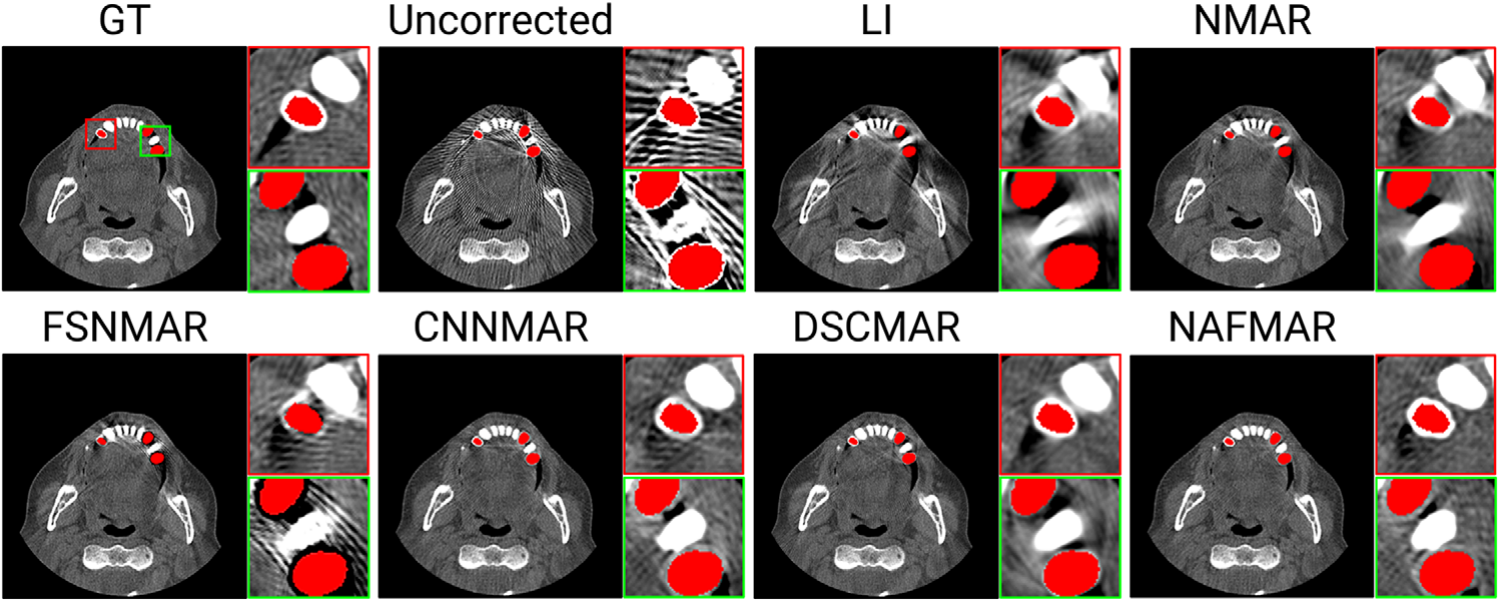
Qualitative comparisons on the simulated dataset with four metal implants. The display window is [−300, 700] Hounsfield unit, and the metal objects are colored red.

**FIGURE 7:**
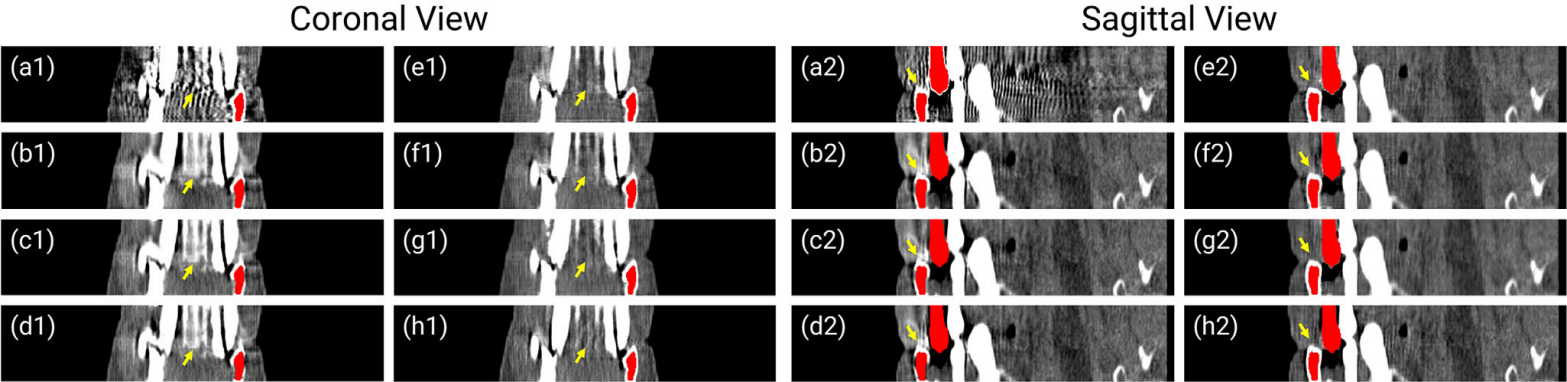
Coronal and sagittal views of reconstructions from the simulated dataset with five metals. (a) Uncorrected. (b) LI. (c) NMAR. (d) FSNMAR. (e) CNNMAR. (f) DSCMAR. (g) NAFMAR. (h) Ground truth. The metal objects are colored red. CNN, convolutional neural network; LI, linear interpolation; MAR, metal artifact reduction; NAF, neural attenuation field.

**FIGURE 8:**
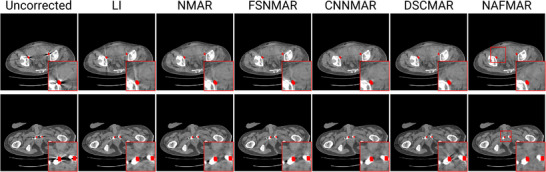
Qualitative comparisons on the clinical dataset. The display window is [‐240, 320] Hounsfield unit, and the metal objects are colored red.

**FIGURE 9:**
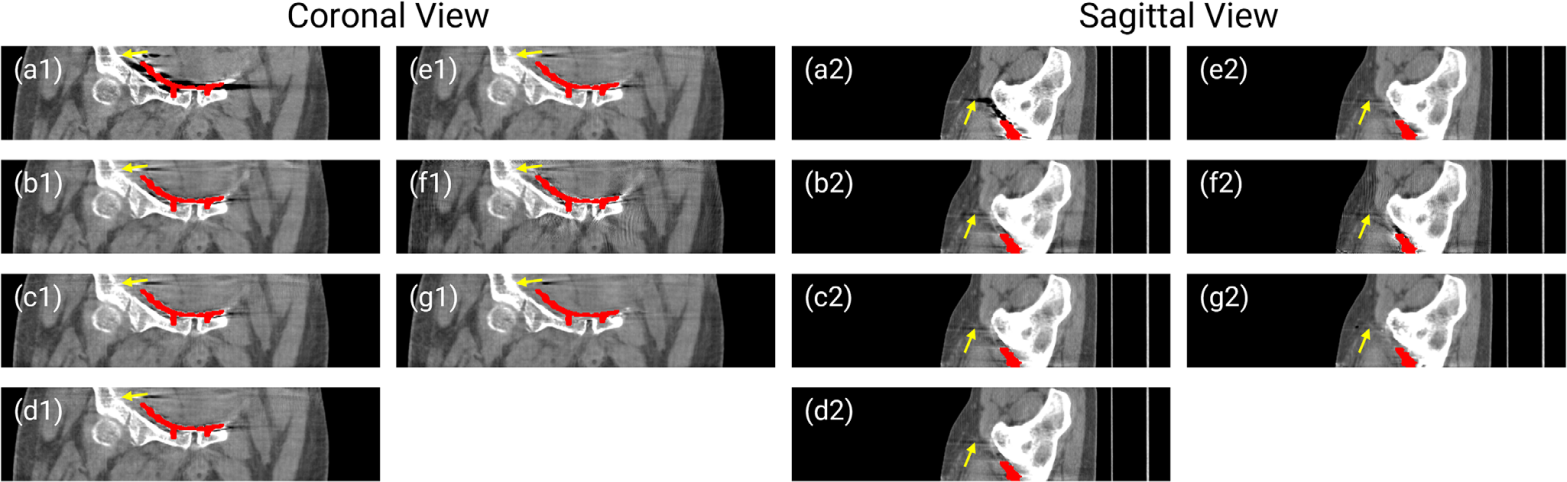
Coronal and sagittal views of reconstructions from the clinical dataset. (a) Uncorrected. (b) LI. (c) NMAR. (d) FSNMAR. (e) CNNMAR. (f) DSCMAR. (g) NAFMAR. The metal objects are colored red. CNN, convolutional neural network; LI, linear interpolation; MAR, metal artifact reduction; NAF, neural attenuation field.

**FIGURE 10:**
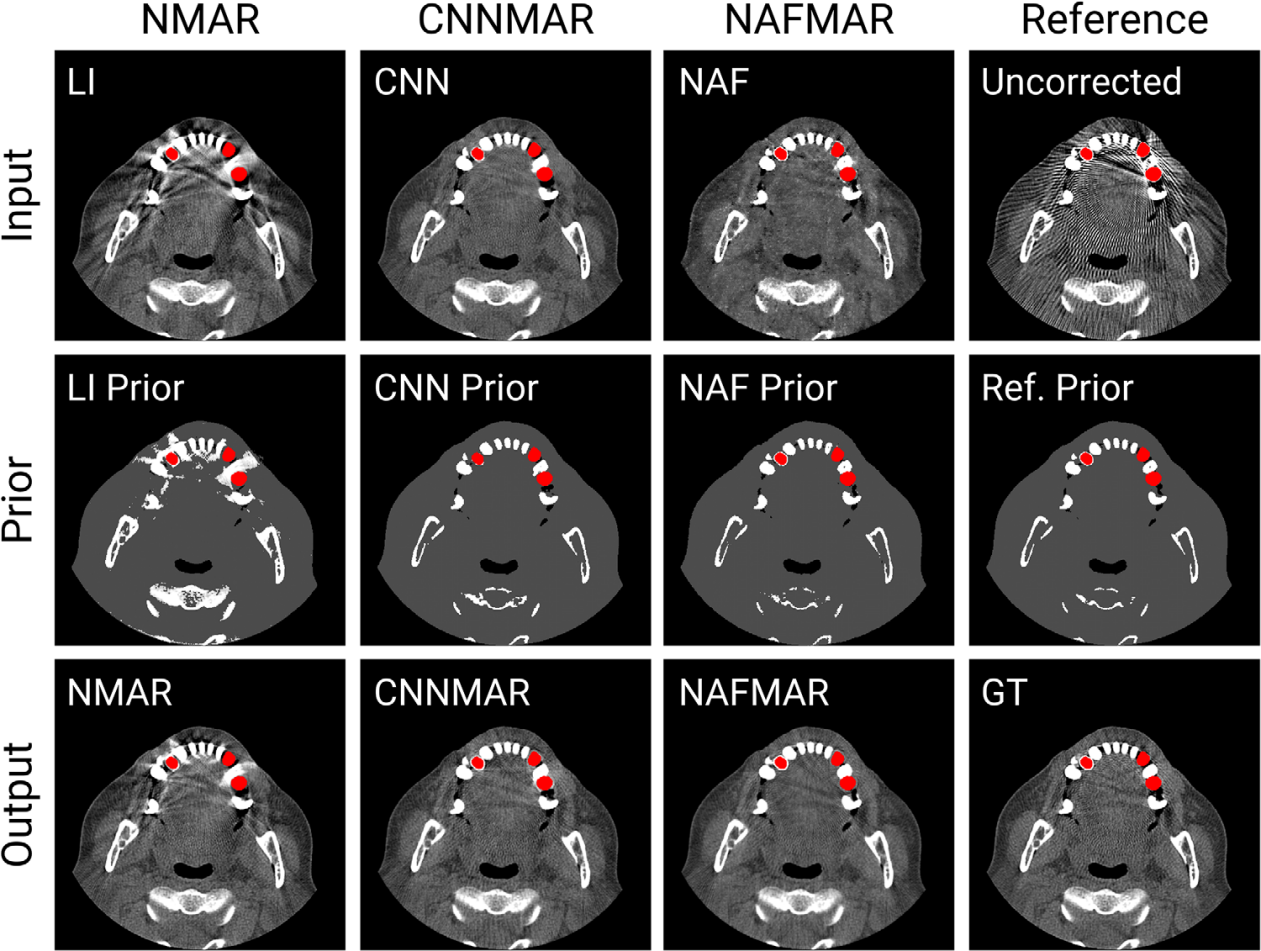
Comparisons of prior images obtained by using LI, CNN, and NAF. The display window is [−300, 700] Hounsfield unit, and the metal objects are colored red. CNN, convolutional neural network; LI, linear interpolation; NAF, neural attenuation field.

**FIGURE 11:**
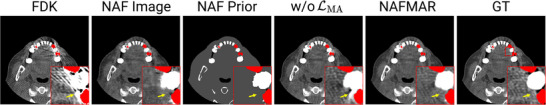
Qualitative ablation study on the effects of sinogram inpainting and metal‐aware loss. The display window is [−300, 700] Hounsfield unit, and the metal objects are colored red.

We quantitatively (Table 1) and qualitatively (Figures 5, 6, 7, 8, 9) demonstrate the effectiveness of the proposed method against the baselines. We then compare prior images obtained by the convolutional neural network and our proposed neural attenuation field (Figure 10) to further understand each method. Finally, we provide ablation studies to validate our design choices in Figure 11.

**TABLE 1:** Quantitative comparisons on the simulated dataset. The best results are indicated in boldface.

	PSNR/SSIM			
	2 metals	3 metals	4 metals	5 metals
Uncorrected	28.51/0.726	28.19/0.702	27.40/0.677	26.38/0.625
LI^7^	29.75/0.832	29.66/0.804	28.55/0.811	28.32/0.795
NMAR^8^	32.38/0.912	32.07/0.890	30.24/0.889	30.26/0.874
FSNMAR^10^	31.54/0.907	31.08/0.883	29.26/0.881	29.48/0.867
CNNMAR^14^	34.35/0.920	34.62/0.916	33.73/0.909	33.23/0.898
DSCMAR^54^	**35.85**/0.922	**35.74**/0.916	**34.43**/0.914	**34.49**/0.904
NAFMAR	35.40/**0.941**	35.54/**0.939**	34.42/**0.931**	33.52/**0.919**

Abbreviations: CNN, convolutional neural network; LI, linear interpolation; MAR, metal artifact reduction; NAF, neural attenuation field; PSNR, peak signal‐to‐noise ratio; SSIM, structural similarity index measure.

### 5.1 Comparisons

In Figures 5 and 6, we present the reconstructions of dental CT images using various MAR methods. As can be observed in the uncorrected images, metal implants cause severe streak artifacts, which degrade image quality and obscure dental structures. While LI and NMAR mitigate these streaks and shadowing artifacts, they compromise anatomical features, particularly near dental fillings, where distortion and secondary artifacts are noticeable. FSNMAR exhibits similar limitations, as it relies on NMAR to produce its final correction. Meanwhile, CNNMAR and DSCMAR show improved artifact reduction by leveraging data‐driven priors. However, closer examination of zoomed‐in images reveals structural loss or distortion. For instance, in the red‐boxed region of Figure 5, both methods fail to reconstruct fine details accurately. In contrast, the proposed NAFMAR more effectively reduces artifacts and restores obscured structures. As shown in the green‐boxed region of Figure 6, NAFMAR reconstructs dental anatomy between implants with high fidelity while producing fewer secondary artifacts compared to DSCMAR. The coronal and sagittal views in Figure 7 further confirm the effectiveness of NAFMAR, as highlighted by the yellow arrows.

This superior performance extends to the clinical dataset, where NAFMAR effectively mitigates metal artifacts. The zoomed‐in images in Figure 8 and the yellow arrows in Figure 9 demonstrate that NAFMAR consistently reduces dark bands and streaks while minimizing secondary artifacts. In contrast, DSCMAR struggles to capture artifacts in the clinical data, primarily due to the limited generalizability of neural networks to unseen data.^62^ Conversely, NAFMAR optimizes a neural attenuation field by enforcing data consistency across 3D geometry, resulting in more stable and superior reconstructions.

The quantitative analysis summarized in Table 1 further supports these findings, where NAFMAR outperforms all baselines in terms of perceptual quality evaluation (i.e., SSIM). Furthermore, it achieves PSNR values comparable to the dual‐domain supervised learning method (i.e., DSCMAR) without the need for large datasets, highlighting the effectiveness of the model‐driven neural field prior for CT metal artifact reduction. Overall, NAFMAR demonstrates high‐fidelity MAR performance, regardless of the number of metallic objects.

### 5.2 Prior images

Next, we compare the prior images generated using LI, CNN, and our proposed NAF method in Figure 10. For reference, the reference prior image was generated from the ground‐truth CT image using the same technique applied for CNN and NAF prior images. Unlike CNN, that requires a large amount of paired CT images to train the network, NAF achieves comparable or even superior prior images without such need for data. Notably, our NAF proves to be a highly effective prior that enables an accurate interpolation along metal traces, as confirmed by the quantitative evaluations in Table 1.

### 5.3 Ablation studies

We validate our algorithm's design choices with an ablation study using our simulated dataset with 4 metal implants. First, we compare the NAF image with the final output, both of which are presented in Figure 11. The NAF image accurately reconstructs the overall structure and effectively reduces streak artifacts. However, it suffers from the loss of textures and residual artifacts persist. In contrast, our method leverages the NAF image to obtain the NAF prior, which is used to inpaint the metal trace. As a result, our method demonstrates high‐fidelity reconstructions that preserve both structural and fine details, as evidenced quantitatively in Table 2.

**TABLE 2:** Quantitative ablation study of our algorithm. The best results are indicated in boldface.

	PSNR	SSIM
FDK	27.40	0.677
NAF image	32.31	0.853
NAF prior	29.89	0.786
NAFMAR without $L_{MA}$	33.89	0.909
NAFMAR (ours)	**34.42**	**0.931**

Abbreviations: FDK, Feldkamp–Kress–Davis algorithm; MAR, metal artifact reduction; NAF, neural attenuation field; PSNR, peak signal‐to‐noise ratio; SSIM, structural similarity index measure.

Next, we analyze the impact of the metal‐aware loss ($L_{\text{MA}}$) on reconstruction quality. As can be seen from the side‐by‐side comparisons in Figure 11, it is apparent that the metal‐aware loss improves the network's ability to accurately capture features near metal. Without the inclusion of metal‐aware loss, protruding artifacts are noticeable in the reconstructed images, as indicated by the yellow arrows. This confirms the effectiveness of our weighting scheme, which adaptively modulates the contribution of sampled rays based on their proximity to metal objects, thereby enhancing our model to learn more informative signals.

## 6 DISCUSSION

### 6.1 Spectral bias

The proposed method of leveraging a neural attenuation field as an image prior demonstrates superior reconstructions compared to other baseline methods. However, it is also important to acknowledge the limitations inherent to neural attenuation fields, particularly in terms of spectral bias, where deep networks are biased toward learning lower frequencies.^31^ Therefore, we further discuss the reconstructions of FDK and NAF in a well‐posed imaging scenario. Here, we used the Mayo Clinic data^63^ to better illustrate the subtle difference in texture and feature. The original image serves as the reference, whereas the FDK and NAF images are reconstructed using simulated 512‐view CBCT projections.

**FIGURE 12:**
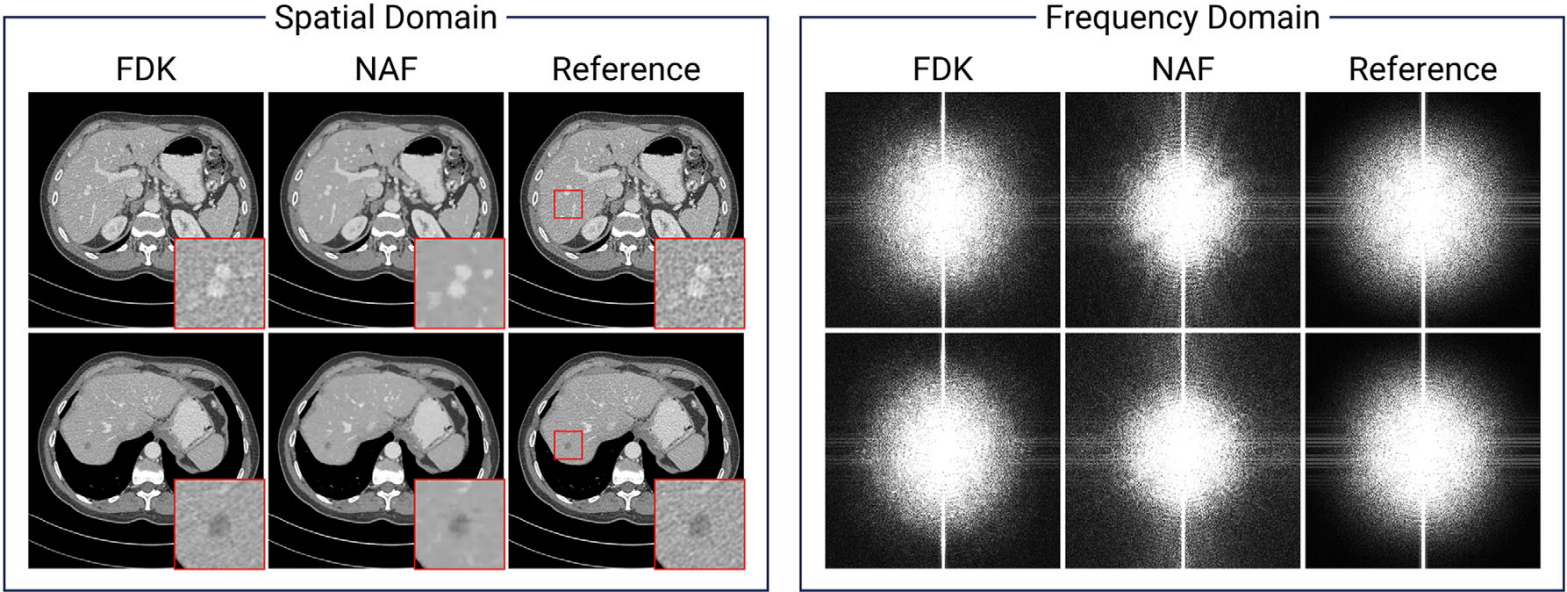
Spectral bias of neural attenuation fields. Full‐view CT reconstructions are visualized in the spatial domain (left) and frequency‐domain magnitude (right). The display window is [‐240 320] Hounsfield unit (left) and consistent across all frequency‐domain magnitude images (right). CT, computed tomography.

Figure 12 compares FDK and NAF reconstructions in both spatial and frequency domains. While FDK reconstruction produces images nearly identical to the reference, NAF struggles to represent high‐frequency variations, such as noise texture. This limitation is evident in the frequency domain, where NAF shows greater discrepancies than FDK. Nevertheless, as highlighted in the red‐boxed images, NAF effectively captures anatomical structures. Therefore, given that the NAF prior image primarily leverages structural information rather than fine details, this further supports the efficacy of our design choice. Note that although techniques like ray‐supersampling^64^ could improve the quality of neural renderings, they often introduce significant increase in training time.

### 6.2 Metal object size

**FIGURE 13:**
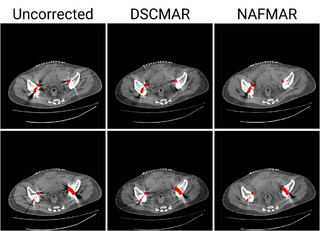
Challenging cases with large metal objects, where metal artifact remains after correction. The display window is [‐240, 320] Hounsfield unit, and the metal objects are colored red.

On the other hand, the size of metal objects affects NAFMAR reconstruction quality. Large metal implants, such as hip prostheses, generate large metal traces that compromise data fidelity. In such cases, methods that rely solely on interpolation (e.g., LI) or lack data‐driven priors (e.g., NMAR and NAFMAR) face inherent difficulties in accurately inpainting extensive metal traces. While NAFMAR has proven effective across varying sizes, shapes, and numbers of metal implants, certain challenging cases remain, particularly those involving large metal objects. As shown in Figure 13, residual artifacts persisted in clinical data after correction. Nonetheless, it is worth noting that even more pronounced residuals are observed in DSCMAR, highlighting the inherent difficulty of metal artifact reduction in extreme cases.

### 6.3 Future work

NAFMAR has demonstrated the potential and effectiveness of leveraging a neural attenuation field prior to address metal artifacts in CT imaging. However, for high‐attenuation metals, increased scatter radiation may present a concern since it can potentially affect regions beyond the metal trace. This limitation is not unique to NAFMAR but is shared by many interpolation‐based approaches, including NMAR and CNNMAR. When scatter‐induced artifacts are significant, scatter correction techniques^65^ could be applied prior to optimizing the neural fields. Alternatively, employing anti‐scatter grids during the scanning process could help reduce scattered photons, thereby improving reconstruction accuracy. Expanding the evaluation of NAFMAR in real clinical settings is an important direction for future work. Investigating its performance in full‐body CT scans would be valuable to assess its robustness a wider range of clinical scenarios.

In addition, future work could explore integrating data‐driven priors into the NAFMAR framework. Leveraging model‐driven and data‐driven approaches in a complementary manner could further enhance the quality of MAR. Moreover, considering the use of plug‐and‐play priors, such as total variation (TV), could refine the neural renderings and contribute to better image quality.

Lastly, from an optimization perspective, enhancing the efficiency of neural field optimization remains a promising direction for further exploration. Currently, NAFMAR requires retraining the model each time new measurements are acquired to obtain patient‐ and system‐specific priors. Recent advancements in neural field optimization, such as meta learning,^66^ sparse voxel grids^67^ or Gaussian representations,^68^ offer promising strategies to reduce training time. Although computational efficiency was not the primary focus of this study, we anticipate that incorporating these techniques could significantly speed up the optimization, thereby realizing NAFMAR's full potential in clinical applications.

## 7 CONCLUSION

In this work, we have proposed NAFMAR, a novel sinogram inpainting method for CBCT metal artifact reduction. Given the corruption of x‐ray projection data induced by metal objects, we have proposed a metal trace‐masked ray sampling strategy that exclusively selects reliable rays to optimize the neural field. Furthermore, the metal‐aware loss was introduced to facilitate the network to prioritize regions near metal implants throughout the optimization, thus enhancing the reconstruction quality. Our experimental evaluation demonstrates the effectiveness of the proposed method of using an NAF prior compared to other prior‐based MAR approaches, including methods relying on extensively pre‐trained neural networks. In summary, NAFMAR provides a high‐fidelity MAR solution for 3D tomographic imaging without requiring a large dataset, which offers great promise for real‐world CBCT applications. We believe that this work will inspire further research in 3D metal artifact reduction, especially exploring the synergies between traditional methods and neural renderings.

## CONFLICT OF INTEREST STATEMENT

The authors declare no conflicts of interest.

## Figures and Tables

**FIGURE A1:**
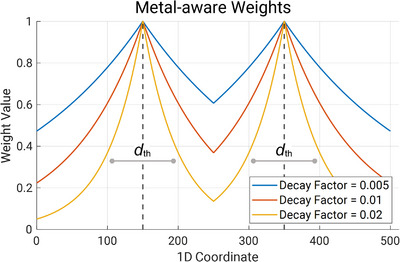
Toy 1D visualizations of metal‐aware weights.

## APPENDIX A

We provide a toy example in Figure A1 to better illustrate the concept of metal‐aware loss. In this example, we assume a single‐detector row CT (e.g., fan‐beam CT), where the x‐axis represents a detector coordinate with a total of 500 cells. The relative metal locations in the detector (i.e., metal traces) are positioned at x=150 and 350, marked by black dashed lines. The metal‐aware weights are then calculated and visualized with varying decay factors of 0.005, 0.01, and 0.02, shown as blue, orange, and yellow lines, respectively. Although this is a 1D example, the cone‐beam CT setup in our study follows a similar approach, considering both x and y directions to account for multi‐row detectors. Here, it is clear that $d_{\text{th}}$ determines the boundary of the metal‐aware region, with higher $d_{\text{th}}$ values increasing the region size. Meanwhile, the decay factor (λ) modulates the contribution of sampled rays based on their distance from the metal objects. In this study, we empirically set $d_{\text{th}}$ to 20 and λ to 0.01 to balance the emphasis on pixels near metal traces, while the method was found to be robust to variations in these  parameters.

## References

[1] Gjesteby L , De Man B , Jin Y , et al. Metal artifact reduction in CT: where are we after four decades? IEEE Access. 2016;4:5826‐5849.

[2] De Man B , Nuyts J , Dupont P , Marchal G , Suetens P . Metal streak artifacts in X‐ray computed tomography: a simulation study. IEEE Trans Nucl Sci. 1999;46:691‐696.

[3] Giantsoudi D , De Man B , Verburg J , et al. Metal artifacts in computed tomography for radiation therapy planning: dosimetric effects and impact of metal artifact reduction. Phys Med Biol. 2017;62:R49‐R80. 28323641 10.1088/1361-6560/aa5293

[4] Wang G , Snyder DL , O'Sullivan JA , Vannier MW . Iterative deblurring for CT metal artifact reduction. IEEE Trans Med Imaging. 1996;15:657‐664. 18215947 10.1109/42.538943

[5] Bal M , Spies L . Metal artifact reduction in CT using tissue‐class modeling and adaptive prefiltering. Med Phys. 2006;33:2852‐2859. 16964861 10.1118/1.2218062

[6] Kyriakou Y , Meyer E , Prell D , Kachelrieß M . Empirical beam hardening correction (EBHC) for CT. Med Phys. 2010;37:5179‐5187. 21089751 10.1118/1.3477088

[7] Kalender WA , Hebel R , Ebersberger J . Reduction of CT artifacts caused by metallic implants. Radiology. 1987;164:576‐577. 3602406 10.1148/radiology.164.2.3602406

[8] Meyer E , Raupach R , Lell M , Schmidt B , Kachelrieß M . Normalized metal artifact reduction (NMAR) in computed tomography. Med Phys. 2010;37:5482‐5493. 21089784 10.1118/1.3484090

[9] Mehranian A , Ay MR , Rahmim A , Zaidi H . X‐ray CT metal artifact reduction using wavelet domain L_{0} sparse regularization. IEEE Trans Med Imaging. 2013;32:1707‐1722. 23744669 10.1109/TMI.2013.2265136

[10] Meyer E , Raupach R , Lell M , Schmidt B , Kachelrieß M . Frequency split metal artifact reduction (FSMAR) in computed tomography. Med Phys. 2012;39:1904‐1916. 22482612 10.1118/1.3691902

[11] LeCun Y , Bengio Y , Hinton G . Deep learning. Nature. 2015;521:436‐444. 26017442 10.1038/nature14539

[12] Wang H , Li Y , He N , Ma K , Meng D , Zheng Y . DICDNet: deep interpretable convolutional dictionary network for metal artifact reduction in CT images. IEEE Trans Med Imaging. 2021;41:869‐880. 10.1109/TMI.2021.3127074 34752391

[13] Kim S , Ahn J , Kim B , Kim C , Baek J . Convolutional neural network–based metal and streak artifacts reduction in dental CT images with sparse‐view sampling scheme. Med Phys. 2022;49:6253‐6277. 35906986 10.1002/mp.15884

[14] Zhang Y , Yu H . Convolutional neural network based metal artifact reduction in x‐ray computed tomography. IEEE Trans Med Imaging. 2018;37:1370‐1381. 29870366 10.1109/TMI.2018.2823083 PMC5998663

[15] Cao W , Parvinian A , Adamo D , et al. Deep convolutional‐neural‐network‐based metal artifact reduction for CT‐guided interventional oncology procedures (MARIO). Med Phys. 2024;51(6):4231‐4242. 38353644 10.1002/mp.16980

[16] Ghani MU , Karl WC . Fast enhanced CT metal artifact reduction using data domain deep learning. IEEE Trans Comput Imaging. 2019;6:181‐193.

[17] Park HS , Lee SM , Kim HP , Seo JK , Chung YE . CT sinogram‐consistency learning for metal‐induced beam hardening correction. Med Phys. 2018;45:5376‐5384. 30238586 10.1002/mp.13199

[18] Wang J , Zhao Y , Noble JH , Dawant BM . Conditional generative adversarial networks for metal artifact reduction in CT images of the ear. In: International Conference on Medical Image Computing and Computer‐Assisted Intervention . Springer; 2018. 10.1007/978-3-030-00928-1_1 PMC6347117 30693351

[19] Lin W‐A , Liao H , Peng C , et al. Dudonet: Dual domain network for ct metal artifact reduction. In: IEEE/CVF Conference on Computer Vision and Pattern Recognition . IEEE; 2019:10504‐10513.

[20] Wang H , Li Y , Zhang H , Meng D , Zheng Y . InDuDoNet+: A deep unfolding dual domain network for metal artifact reduction in CT images. Med Image Anal. 2023;85:102729. 36623381 10.1016/j.media.2022.102729

[21] Lee J , Gu J , Ye JC . Unsupervised CT metal artifact learning using attention‐guided β‐CycleGAN. IEEE Trans Med Imaging. 2021;40:3932‐3944. 34329157 10.1109/TMI.2021.3101363

[22] Liu X , Xie Y , Diao S , Tan S , Liang X . Unsupervised CT metal artifact reduction by plugging diffusion priors in dual domains. IEEE Trans Med Imaging. 2024;43:3533‐3545. 38194400 10.1109/TMI.2024.3351201

[23] Paschali M , Conjeti S , Navarro F , Navab N . Generalizability vs. robustness: investigating medical imaging networks using adversarial examples. In: International Conference on Medical Image Computing and Computer‐Assisted Intervention . Springer; 2018.

[24] Kim S , Kim B , Lee J , Baek J . Sparsier2Sparse: self‐supervised convolutional neural network‐based streak artifacts reduction in sparse‐view CT images. Med Phys. 2023;50(12):7731‐7747. 37303108 10.1002/mp.16552

[25] Kim B , Shim H , Baek J . A streak artifact reduction algorithm in sparse‐view CT using a self‐supervised neural representation. Med Phys. 2022;49:7497‐7515. 35880806 10.1002/mp.15885

[26] Mildenhall B , Srinivasan PP , Tancik M , Barron JT , Ramamoorthi R , Ng R . NeRF: representing scenes as neural radiance fields for view synthesis. Commun. ACM, 2020;95:99‐106.

[27] Zang G , Idoughi R , Li R , Wonka P , Heidrich W . Intratomo: self‐supervised learning‐based tomography via sinogram synthesis and prediction. In: IEEE/CVF International Conference on Computer Vision . IEEE; 2021:1940‐1950.

[28] Lee J , Baek J . Iterative reconstruction for limited‐angle CT using implicit neural representation. Phys Med Biol. 2024;69:105008. 10.1088/1361-6560/ad3c8e 38593820

[29] Zha R , Zhang Y , Li H . NAF: neural attenuation fields for sparse‐view CBCT reconstruction. In: International Conference on Medical Image Computing and Computer‐Assisted Intervention . Springer; 2022.

[30] Rückert D , Wang Y , Li R , Idoughi R , Heidrich W . NeAT: neural adaptive tomography. ACM Trans Graph (TOG). 2022;41:1‐13.

[31] Rahaman N , Baratin A , Arpit D , et al. On the spectral bias of neural networks. In International Conference on Machine Learning . PLMR; 2019:5301‐5310.

[32] Zhang Y , Yan H , Jia X , Yang J , Jiang SB , Mou X . A hybrid metal artifact reduction algorithm for x‐ray CT. Med Phys. 2013;40:041910. 23556904 10.1118/1.4794474

[33] De Man B , Nuyts J , Dupont P , Marchal G , Suetens P . An iterative maximum‐likelihood polychromatic algorithm for CT. IEEE Trans Med Imaging. 2001;20:999‐1008. 11686446 10.1109/42.959297

[34] Karageorgos GM , Zhang J , Peters N , et al. A denoising diffusion probabilistic model for metal artifact reduction in CT. IEEE Trans Med Imaging. 2024;43:3521‐3532. 38963746 10.1109/TMI.2024.3416398 PMC11657996

[35] Fan F , Ritschl L , Beister M , et al. Simulation‐driven training of vision transformers enables metal artifact reduction of highly truncated CBCT scans. Med Phys. 2024;51:3360‐3375. 38150576 10.1002/mp.16919

[36] Wang H , Xie Q , Zeng D , Ma J , Meng D , Zheng Y . OSCNet: orientation‐shared convolutional network for CT metal artifact learning. IEEE Trans Med Imaging. 2023;43:489‐502. 10.1109/TMI.2023.3310987 37656650

[37] Liao H , Lin W‐A , Zhou SK , Luo J . ADN: artifact disentanglement network for unsupervised metal artifact reduction. IEEE Trans Med Imaging. 2019;39:634‐643. 31395543 10.1109/TMI.2019.2933425

[38] Zhou B , Chen X , Zhou SK , Duncan JS , Liu C . DuDoDR‐Net: dual‐domain data consistent recurrent network for simultaneous sparse view and metal artifact reduction in computed tomography. Med Image Anal. 2022;75:102289. 34758443 10.1016/j.media.2021.102289 PMC8678361

[39] Szegedy C , Zaremba W , Sutskever I , et al. Intriguing properties of neural networks. arXiv preprint arXiv:1312.6199 (2013).

[40] Tewari A , Thies J , Mildenhall B , et al. Advances in neural rendering. Comput Graph Forum. 2022;41:703‐735.

[41] Yang J , Pavone M , Wang Y . FreeNeRF: improving few‐shot neural rendering with free frequency regularization. In: IEEE/CVF Conference on Computer Vision and Pattern Recognition , IEEE; 2023.

[42] Sitzmann V , Martel J , Bergman A , Lindell D , Wetzstein G . Implicit neural representations with periodic activation functions. Adv Neural Inf Process Syst. 2020;33:7462‐7473.

[43] Li Z , Müller T , Evans A , et al. Neuralangelo: high‐fidelity neural surface reconstruction. In: IEEE/CVF Conference on Computer Vision and Pattern Recognition . IEEE; 2023.

[44] Cai Y , Wang J , Yuille A , Zhou Z , Wang A . Structure‐aware sparse‐view x‐ray 3D reconstruction. In: IEEE/CVF Conference on Computer Vision and Pattern Recognition , IEEE; 2024.

[45] Wu Q , Chen L , Wang C , et al. Unsupervised polychromatic neural representation for ct metal artifact reduction. Adv Neural Inf Process Syst. 2023;36:69605‐69624.

[46] Leinweber C , Maier J , Kachelrieß M . X‐ray spectrum estimation for accurate attenuation simulation. Med Phys. 2017;44:6183‐6194. 28975632 10.1002/mp.12607

[47] Lee J , Ahn J , Baek J . Neural attenuation fields for metal artifact reduction in dental CT. In: Medical Imaging 2024: Physics of Medical Imaging. Vol 12925. SPIE; 2024:173‐179.

[48] Müller T , Evans A , Schied C , Keller A . Instant neural graphics primitives with a multiresolution hash encoding. ACM Trans Graph (ToG). 2022;41:1‐15.

[49] Gai Z , Liu Z , Tan M , et al. EGRA‐NeRF: edge‐guided ray allocation for neural radiance fields. Image Vision Comput. 2023;134:104670.

[50] Kheradmand S , Rebain D , Sharma G , et al. Accelerating neural field training via soft mining. In: IEEE/CVF Conference on Computer Vision and Pattern Recognition . IEEE; 2024:20071‐20080.

[51] Huang X , Li W , Hu J , Chen H , Wang Y . RefSR‐NeRF: towards high fidelity and super resolution view synthesis. In: IEEE/CVF Conference on Computer Vision and Pattern Recognition . IEEE; 2023:8244‐8253.

[52] Feldkamp LA , Davis LC , Kress JW . Practical cone‐beam algorithm. J Opt Soc Am A. 1984;1:612‐619.

[53] Clark K , Vendt B , Smith K , et al. The cancer imaging archive (TCIA): maintaining and operating a public information repository. J Digit Imaging. 2013;26:1045‐1057. 23884657 10.1007/s10278-013-9622-7 PMC3824915

[54] Yu L , Zhang Z , Li X , Xing L . Deep sinogram completion with image prior for metal artifact reduction in CT images. IEEE Trans Med Imaging. 2020;40:228‐238. 32956044 10.1109/TMI.2020.3025064 PMC7875504

[55] Herman GT . Correction for beam hardening in computed tomography. Phys Med Biol. 1979;24:81‐106. 432276 10.1088/0031-9155/24/1/008

[56] Hubbell JH , Seltzer SM . Tables of X‐ray mass attenuation coefficients and mass energy‐absorption coefficients 1 keV to 20 MeV for elements Z= 1 to 92 and 48 additional substances of dosimetric interest. Technical report. National Institute of Standards and Technology; 1995.

[57] Liu P , Han H , Du Y , et al. Deep learning to segment pelvic bones: large‐scale CT datasets and baseline models. Int J Comput Assist Radiol Surg. 2021;16:749‐756. 33864189 10.1007/s11548-021-02363-8

[58] Kirillov A , Mintun E , Ravi N , et al. Segment anything. In: IEEE/CVF International Conference on Computer Vision , IEEE; 2023.

[59] Wang Z , Bovik AC , Sheikh HR , Simoncelli EP . Image quality assessment: from error visibility to structural similarity. IEEE Trans Image Process. 2004;13:600‐612. 15376593 10.1109/tip.2003.819861

[60] Kingma DP , Ba J . Adam: a method for stochastic optimization. arXiv preprint arXiv:1412.6980 (2014).

[61] Biguri A , Dosanjh M , Hancock S , Soleimani M . TIGRE: a MATLAB‐GPU toolbox for CBCT image reconstruction. Biomed Phys Eng Express. 2016;2:055010.

[62] Krois J , Garcia Cantu A , Chaurasia A , et al. Generalizability of deep learning models for dental image analysis. Sci Rep. 2021;11:6102. 33731732 10.1038/s41598-021-85454-5 PMC7969919

[63] Moen TR , Chen B , Holmes III DR , et al. Low‐dose CT image and projection dataset. Med Phys. 2021;48:902‐911. 33202055 10.1002/mp.14594 PMC7985836

[64] Wang C , Wu X , Guo Y‐C , Zhang S‐H , Tai Y‐W , Hu S‐M . NeRF‐SR: high quality neural radiance fields using supersampling. In: ACM International Conference on Multimedia . Association for Computing Machinery; 2022:6445‐6454.

[65] Trapp P , Maier J , Susenburger M , Sawall S , Kachelrieß M . Empirical scatter correction: CBCT scatter artifact reduction without prior information. Med Phys. 2022;49:4566‐4584. 35390181 10.1002/mp.15656

[66] Tancik M , Mildenhall B , Wang T , et al. Learned initializations for optimizing coordinate‐based neural representations. In: IEEE/CVF Conference on Computer Vision and Pattern Recognition . IEEE; 2021:2846‐2855.

[67] Fridovich‐Keil S , Yu A , Tancik M , Chen Q , Recht B , Kanazawa A . Plenoxels: radiance fields without neural networks. In: IEEE/CVF Conference on Computer Vision and Pattern Recognition . IEEE, 2022:5501‐5510.

[68] Kerbl B , Kopanas G , Leimkühler T , Drettakis G . 3D Gaussian splatting for real‐time radiance field rendering. ACM Trans Graph. 2023;42:1‐14.

